# Complete Genome Sequence of Subcluster 5.2 *Synechococcus* sp. Strain CB0101, Isolated from the Chesapeake Bay

**DOI:** 10.1128/MRA.00484-19

**Published:** 2019-08-29

**Authors:** Daniel Fucich, David Marsan, Ana Sosa, Feng Chen

**Affiliations:** a The Institute of Marine and Environmental Technology, University of Maryland Center for Environmental Science, Baltimore, Maryland, USA; Indiana University, Bloomington

## Abstract

*Synechococcus* sp. strain CB0101 is a model strain for cyanobacteria living in the estuarine environment. It is also a representative member of marine *Synechococcus* subcluster 5.2. The draft genome sequence of CB0101 was reported in 2014 with 454 sequencing. Here, we report the complete genome sequence of CB0101, obtained with PacBio sequencing. CB0101 contains a specialized array of genes which are involved in sensing, responding to, and persisting in the presence of environmental stress.

## ANNOUNCEMENT

*Synechococcus* is the ubiquitous genus of the picocyanobacteria. *Synechococcus* spp. contribute significantly to global primary productivity due to their high abundance (1). Many genomes of marine *Synechococcus* spp. have been reported (2–4), but the majority of them are coastal and oceanic *Synechococcus* spp., and few are from estuaries. It has been reported that a unique genotype and ecotype of *Synechococcus* are present in the Chesapeake Bay estuary (5–7). *Synechococcus* sp. strain CB0101 was isolated from the Chesapeake Bay with preincubation and pour plating using SN medium with adjusted salinity to match water samples described by Chen et al. (5). *Synechococcus* sp. CB0101 was grown in the SN medium at 25°C in constant light (20 to 30 μE m^−2^ s^−1^) in an illuminated incubator. *Synechococcus* CB0101 is a representative strain of estuarine *Synechococcus* spp. which belongs to subcluster 5.2 (8, 9). CB0101 has shown much higher tolerance to temperature, salinity, and heavy metals than have coastal and oceanic *Synechococcu*s strains (10). It appears that estuarine *Synechococcus* strains exhibit unique ecophysiological features that enable them to adapt to highly dynamic environments like the Chesapeake Bay. Because of its ecological relevance, the draft genome sequence of CB0101 was reported by Marsan et al. in 2014 (8). The draft genome sequence of CB0101 is 2,686,395 bp long, with 3,109 genes and 46 tRNAs and 15 total rRNA gene copies. Annotation yielded 3 5S rRNAs, 7 23S rRNAs, and 5 16S rRNAs. In order to fully understand the molecular mechanisms in response to environmental changes, we began to apply different omics (i.e., transcriptomics and proteomics) to the study of CB0101. We quickly noticed the need to obtain a complete genome sequence of CB0101.

The complete genome sequence of CB0101 was determined using a hybrid approach combining 454 GS-FLX Titanium 8-kb paired-end and PacBio libraries. Cells were collected using centrifugation (10,000 × *g*, 10 min), and the pellet was transferred into a 2-ml tube and processed immediately. We obtained DNA from 25-ml cultures using the Epicentre MasterPure kit. The first round of sequencing was completed using a 454 GS FLX machine for whole-genome sequencing with the source being genomic. Selection was random with a paired-read layout. This yielded 439,486 reads with an average length of 445 bp. Unfortunately, only a draft genome could be completed using this approach, so to amend this and close the genome, PacBio sequencing at the Institute of Genome Sciences (IGS) obtained 121,716 total reads with an average read length of 6,295 bp, resulting in ∼40× coverage on the genome. Parameters used for PacBio sequencing included a long library type (5 to 20 kb, polymerase P6, C4 sequencing chemistry, and a movie length of 240 min). The PacBio sequencing greatly improved genome assembly compared to the previous 454 sequencing run (8). Low-quality regions of sequencing data were removed from the raw PacBio reads using the Hierarchical Genome Assembly Process (HGAP) v2.2 assembly program, with default parameters (11). Contigs were ordered into putative scaffolds based on their similarity to closely related closed *Synechococcus* genomes, as determined using Mauve (12). As the cultures sequenced were known to contain heterotrophs, we identified the most “*Synechococcus*-like” contigs from nonaxenic cultures by searching each resulting contig against a custom database of sequenced marine microbial genomes using BLAST (13). Contigs with a best match to a non-*Synechococcus* genome were removed from the assembly. The remaining contigs, from both the 454 and PacBio sequencing runs, were combined and assembled using the Sequencher v5.3 software (Gene Codes Corporation, Ann Arbor, MI, USA). The combination of PacBio and 454 sequencing enabled us to assemble a complete genome of CB0101.

**FIG 1:**
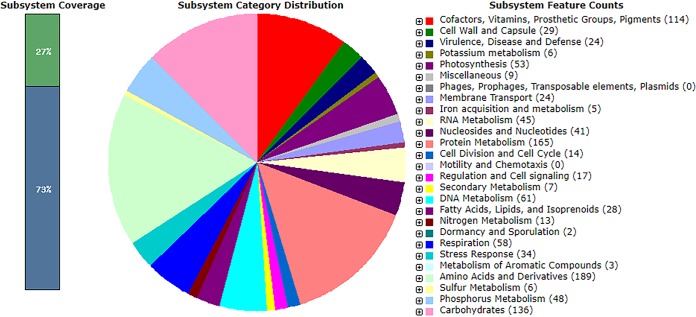
Subsystem coverage and distribution of *Synechococcus* sp. strain CB0101 (based on RAST annotation).

The complete genome of CB0101 consists of 2,789,657 bp (64.1% G+C content), with 3,128 genes, 52 tRNAs, and 24 rRNAs. The final assembly of CB0101 was annotated using the Rapid Annotations using Subsystems Technology (RAST) server (14–16). CB0101 contains many unique genes, as nearly half (46%) of the coding sequences (CDS) were hypothetical proteins. About 73% of the CDS (2,304 genes) were not grouped in the RAST functional subsystems (Fig. 1). CB0101 contains genes associated with an increased capacity to sense and respond to changes in the environment, including 24 membrane transporter genes, 17 regulation and cell signaling genes, 34 stress response genes, 24 virulence, disease, and defense genes, and 2 dormancy genes. Seven pairs of toxin-antitoxin (TA) genes were identified in the CB0101 genome (9, 10). TA genes are not commonly seen in *Synechococcus* spp. living in coastal and oceanic water (10). It has been shown that some TA genes are actively expressed under oxidative stress caused by zinc toxicity or high light exposure (9). Information gleaned from the complete genome showed the ability of CB0101 to sense and respond to environmental conditions, such as nitrogen or phosphate depletion and zinc metal excess. *Synechococcus* CB0101 contains specialized genes for persisting in the presence of highly dynamic environmental stressors and can be used to further study the molecular ecology of estuarine *Synechococcus* spp.

### Data availability.

This complete genome sequencing project has been submitted to the NCBI under the accession number CP039373. The raw sequence reads for both the 454 GS-FLX Titanium 8-kb paired-end and PacBio libraries are available at accession numbers SRX018027 and PRJNA529695, respectively.
